# Negative Effects of Rhizobacteria Association on Plant Recruitment of Generalist Predators

**DOI:** 10.3390/plants11070920

**Published:** 2022-03-29

**Authors:** Tobias B. Löser, Dani Lucas-Barbosa, Monika Maurhofer, Mark C. Mescher, Consuelo M. De Moraes

**Affiliations:** 1 Department of Environmental System Sciences, ETH Zürich, 8092 Zürich, Switzerland; tobias.loeser@gmail.com (T.B.L.); dani.lucas-barbosa@vetparas.uzh.ch (D.L.-B.); monika.maurhofer@usys.ethz.ch (M.M.); mescher@usys.ethz.ch (M.C.M.); 2 Vector Entomology Group, Institute of Parasitology, Vetsuisse Faculty, University of Zürich, 8057 Zürich, Switzerland

**Keywords:** direct resistance, indirect resistance, *Pseudomonas*, *Spodoptera littoralis*, *Podisus maculiventris*, tomato plants, plant volatiles

## Abstract

Plant-associated microbes can influence above- and belowground interactions between plants and other organisms and thus have significant potential for use in the management of agricultural ecosystems. However, fully realizing this potential will require improved understanding of the specific ways in which microbes influence plant ecology, which are both more complex and less well studied than the direct effects of microbes on host-plant physiology. Microbial effects on mutualistic and antagonistic interactions between plants and insects are of particular interest in this regard. This study examines the effects of two strains of *Pseudomonas* rhizobacteria on the direct and indirect (predator-mediated) resistance of tomato plants to a generalist herbivore (*Spodoptera littoralis*) and associated changes in levels of defense compounds. We observed no significant effects of rhizobacteria inoculation on caterpillar weight, suggesting that rhizobacteria did not influence direct resistance. However, the generalist predator *Podisus maculiventris* avoided plants inoculated with one of our rhizobacteria strains, *Pseudomonas simiae*. Consistent with these results, we found that inoculation with *P. simiae* influenced plant volatile emissions, but not levels of defense-related compounds. These findings show that rhizobacteria can negatively affect the attraction of generalist predators, while highlighting the complexity and context dependence of microbial effects on plant–insect interactions.

## 1. Introduction

Plant-associated microbiomes have profound and wide-ranging effects on plant phenotypes [1,2,3,4]. In addition to effects on plant nutrition, growth, and development, microbes can influence interactions among plants, and between plants and other organisms, with implications for plant fitness and the functioning of natural and agricultural ecosystems [5,6,7]. There is considerable interest in using microbes to steer such interactions to the benefit of the plant by strengthening plant resistance to pests while enhancing interactions with beneficial insects [5,8,9,10]. However, we currently have only a limited understanding of microbe-mediated effects on ecological interactions.

Bacteria of the genus *Pseudomonas* are prominent members of the plant rhizosphere microbiome and have been isolated from roots of many different crop plants [11,12,13]. Plant-associated pseudomonads are widely used in agriculture because of their plant-beneficial properties [8], including antagonistic activity against pathogens [14]. Recently, their effects on plant–insect interactions have received increased attention, and several *Pseudomonas* strains have been found to enhance direct defenses against insect herbivores via plant defense priming [15,16]. Pseudomonads have also been shown to affect tritrophic interactions among plants, herbivores, and herbivores’ natural enemies [17,18], indicating that these rhizobacteria might influence—and potentially enhance—indirect (i.e., natural-enemy mediated) plant defenses, such as herbivore-induced volatile emissions that attract parasitoids and predators [19].

While the detailed mechanisms underlying such effects are not well understood, changes in plant volatile emissions might be caused by microbial effects on plant nutrient acquisition or plant defense signaling. For example, negative effects on the recruitment of an aphid parasitoid to aphid-infested plants via changes in plant volatile emissions was shown to depend on rhizobacteria-mediated changes in jasmonic acid (JA)-signaling [18]. Moreover, plant association with mycorrhiza has been shown to alter plant volatile emissions by effects on plant signaling pathways or phosphate acquisition, potentially affecting the attraction of beneficial insects, such as bio-control agents of the herbivores [20,21]. 

The goal of the present study was to address whether inoculation with rhizobacteria affects antagonistic and mutualistic interactions of plants with insects. To achieve this, we tested the effects of inoculation with two *Pseudomonas* strains on the direct and indirect defenses of tomato plants against the generalist herbivore *Spodoptera littoralis* and assessed associated changes in phytohormones and key metabolites associated with plant resistance, including herbivore-induced plant volatiles. Both *Pseudomonas* strains used here have previously been shown to induce systemic resistance against pathogens in tomato [22,23], but their ability to alter direct and indirect defenses against *S. littoralis* has not previously been explored. So far, *Pseudomonas*-plant–herbivore interactions have been mainly studied with the model plant *Arabidopsis*. For this study, we have chosen tomato in order to study these interactions on an agriculturally relevant crop plant.

## 2. Results

### 2.1. Inoculation with Rhizobacteria Did Not Affect Plant Biomass or Direct Resistance in Tomato

**Figure 1:**
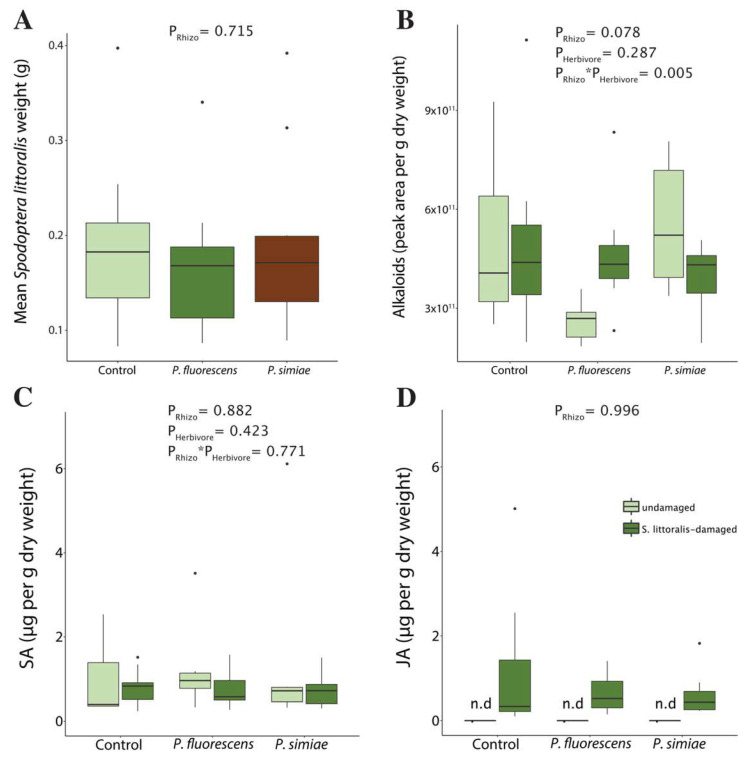
Caterpillar biomass, alkaloids, and phytohormones in tomato plants *Solanum lycopersicum* exposed to inoculation with one of two strains of rhizobacteria and herbivory by *Spodoptera littoralis*. (**A**) Biomass of *Spodoptera littoralis* larvae after a 7-day feeding period. (**B**) Levels of foliar alkaloids, (**C**) salicylic acid (SA) and (**D**) jasmonic acid (JA) in undamaged and *S. littoralis*-damaged leaflets of the respective treatments. Data comprises 10 biological replicates for the performance assay with *S. littoralis* and 5–12 replicates for the defensive metabolites. n.d. = not detected.

We observed no effect of inoculation with *P. simiae* (generalized linear mixed model, *P* = 0.861) or *P. fluorescens* (generalized linear mixed model, *P* = 0.710) on plant biomass (Figure S1). Furthermore, the biomass of caterpillars reared on plants inoculated with each of the rhizobacteria strains was similar to those reared on control plants (generalized linear model, *P* = 0.715) (Figure 1A). Herbivory by *S. littoralis* resulted in a de novo production of JA, but had no effect on salicylic acid (SA) (ANOVA, *P* = 0.423) or alkaloid levels (generalized least-squares model, *P* = 0.287). Inoculation with rhizobacteria had no effect on SA (ANOVA, *P* = 0.882) or JA concentrations (ANOVA, *P* = 0.996) in either undamaged or *S. littoralis*-damaged plants (Figure 1B–D). Similarly, rhizobacteria had no effect on alkaloid content (generalized least-squares model, *P* = 0.078), though we observed a trend towards a lower alkaloid content in undamaged plants inoculated with *P. fluorescens* and a significant interaction between the factors rhizobacterium and herbivory.

### 2.2. Inoculation with P. simiae Renders Tomato Plants Less Attractive to the Predator P. maculiventris

**Figure 2:**
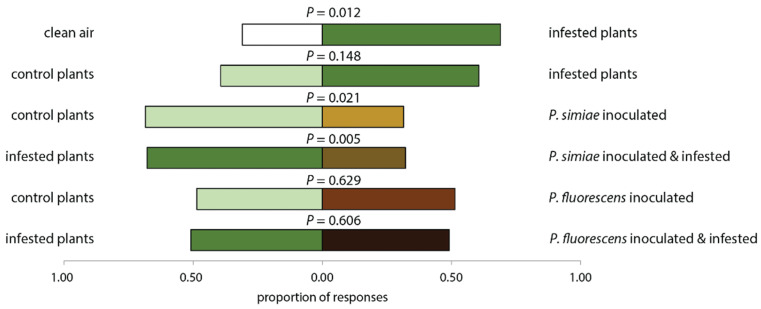
Proportion of spined soldier bug *Podisus maculiventris* responding to odor sources in pairwise choice assays. Tomato plants (*Solanum lycopersicum*) were exposed to inoculation with one of two strains of rhizobacteria and herbivory by *Spodoptera littoralis.* 5–10 bugs were tested per pair of plant with 6–8 replicates of each plant pair. Pairwise comparison of treatments with binomial test.

Inoculation with *P. simiae* reduced the attraction of the soldier bug *P. maculiventris* to both infested (Figure 2, Binomial test, *P* = 0.005) and uninfested plants (Figure 2, Binomial test, *P* = 0.021). In contrast, inoculation with *P. fluorescens* did not influence the attraction of *P. maculiventris* to tomato plants, whether plants were infested with caterpillars of *S. litorallis* or not (Figure 2). We observed no preference of bugs between infested- and uninfested-control plants (Figure 2, Binomial test, *P* = 0.148).

**Figure 3:**
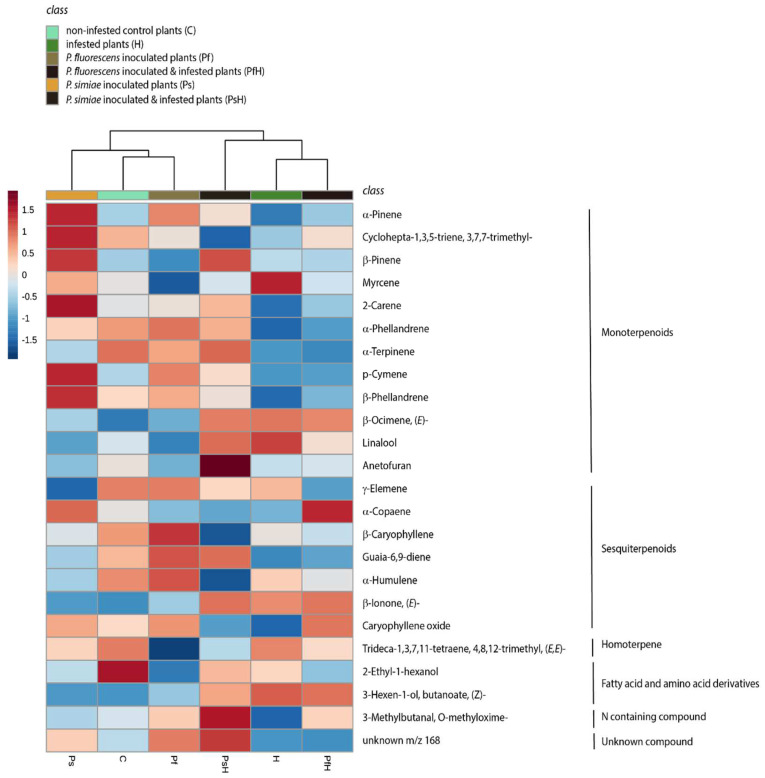
Dendrogram and heatmap of the emission of volatile compounds by tomato plants *Solanum lycopersicum* exposed to inoculation with one of two strains of rhizobacteria and herbivory by *Spodoptera littoralis*. Dendrogram clustering was performed using Ward’s clustering algorithm with Euclidean distances (peak height/g FW) for each compound. Each treatment had 15 replicates.

The odor profile of tomato plants comprised 25 compounds, including 15 monoterpenoids, 7 sesquiterpenoids, the homoterpene (*E,E*)-Trideca-1,3,7,11-tetraene, 4,8,12-trimethyl, and the nitrogen containing compound, O-methyloxime-3-methylbutanal, along with two fatty-acid/amino-acid derivatives (Table S1). Clustering analyses show that treatments in which plants were exposed to herbivory grouped together, while control plants grouped with *P. fluorescens*-inoculated plants (Figure 2). Moreover, clustering analyses suggest an overall higher emission of monoterpenoids in plants inoculated with *P. simiae* (Figure 3).

## 3. Discussion

Our results reveal that association with a rhizobacterium can negatively influence indirect plant resistance against herbivory via effects on predator recruitment. In Y-tube olfactometer assays, tomato plants inoculated with *P. simiae* were significantly less attractive to the generalist predator *Podisus maculiventris* irrespective of whether plants were damaged by the herbivore *Spodoptera littoralis* or undamaged. However, this effect was specific to *P. simiae*, as no similar effect was observed for *P. fluorescens*. Meanwhile, neither of these strains had significant effects on the direct defense traits we measured, including levels of key phytohormones (JA and SA) and alkaloid compounds.

The observation that *P. maculiventris* soldier bugs avoid plants inoculated with *P. simiae* complements previous work showing that soil-borne microbes can influence the preferences of parasitoids [10,17,18,24] and predators [25,26,27]. Previous studies with *P. simiae* in *Arabidopsis thaliana* reported contrasting effects on indirect plant defenses. In one study, inoculation with *P. simiae* rendered *A. thaliana* plants infested with the generalist chewing herbivore *Mamestra brassicae* more attractive to the parasitoid *Microplitis mediator* [17]. However, another study reported that the same strain rendered aphid-infested plants less attractive to the aphid parasitoid *Diaeretiella rapae* [18]. Together with these previous observations, the current results suggest that individual rhizobacteria strains can have contrasting effects on indirect plant defenses depending on the specific ecological context.

The observed effect of *P. simiae*, but not *P. fluorescens*, on predator behavior is consistent with patterns observed in our volatile data, where cluster analyses revealed that the emission profiles of *P. simiae*-inoculated plants diverged from those of *P. fluorescens*-inoculated and control plants, which were more similar to one another. These analyses also suggested overall higher emissions of monoterpenes in *P. simiae*-inoculated plants, consistent with previous reports that inoculation with *P. simiae* led to higher emissions of monoterpenes and increased the monoterpene content in essential oils of peppermint plants [28,29]. Whether effects of *P. simiae* on monoterpene emissions explain the observed effects on *P. maculiventris* attraction remains to be seen; however, it bears noting that herbivory by *S. littoralis* larvae in our experiments had somewhat similar effects on monoterpene emissions with no apparent effects on *P. maculiventris* recruitment.

Previous electroantennogram (EAGs) experiments revealed that *P. maculiventris* perceives a range of plant volatiles [30,31,32], although the importance of odor cues for prey location by this species remains uncertain [33]. In the current study, *P. maculiventris* exhibited a clear preference for plant odors over clean air but did not discriminate between undamaged and herbivore-damaged plants. The aggregation pheromone of *P. maculiventris* contains monoterpenes that elicit EAG responses, indicating that *P. maculiventris* is sensitive to compounds of this class [30,31,34]. These bugs may also be sensitive to cues associated with changes in plant nutritional quality, as *Podisus maculiventris* is also known to feed on plant sap, especially when prey is scarce, and utilization of this resource has been shown to improve its fitness via increased survival rates, reduced development time, and larger body size [35]. It is thus possible that *P. simiae*-induced nutritional changes in tomato plants are reflected in volatile emissions in a way that indicates an inferior host plant quality to *P. maculiventris*. Indeed, changes in nutrient availability by different fertilizer regimes can change plant volatile emissions [36,37,38], and a recent study found that tomato plants inoculated with *P. simiae* differed in soluble sugar, nitrogen, and carbon levels when compared to mock-inoculated control plants [39].

Relevant changes in plant odors might also be caused by microbe-induced changes in plant defense chemistry. In the current study, no significant effects on the performance of *S. littoralis* larvae were quantified. A recent study in *A. thaliana* reported contrasting effects showing enhanced resistance to a pathogen but decreased resistance to *S. littoralis* following root-colonization by a *Pseudomonas* strain [40]. These findings suggest that rhizobacteria-induced resistance to pathogens may involve different mechanisms than those that underly resistance to *S. littoralis*. As the current results reveal no significant effects of either rhizobacteria strain on defense-related phytohormones or defensive compounds (alkaloids), we speculate that *P. simiae* may affect plant resource allocation, which could influence plant volatiles and the associated behavioral preferences of *P. maculiventris*. This could be further investigated in studies that include the reproductive stage and estimate seed production.

Plant-associated rhizobacteria can alter plant traits in ways that influence their interactions with other organisms [3,4,10], yet few studies explore direct and indirect defenses when investigating the interactions with herbivores. The current findings show that plant-associated rhizobacteria can influence tritrophic interactions. Specifically, we found that inoculation with *P. simiae* renders tomato plants less attractive to the generalist predator *P. maculiventris*, which could increase the susceptibility of agricultural crops to herbivory. Together with previous results, the current findings also highlight the variability and context dependence of rhizobacteria-mediated effects on plant direct and indirect resistance to herbivory. Not only can different rhizobacteria strains have different effects on the same interactions, but the same strain may also have divergent effects across different ecological settings. Meanwhile, our understanding of the specific mechanisms underlying these variable outcomes remains remarkably limited. We suggest that future studies on the use of resistance and growth promoting microbial agents should account for potential effects of microbial inoculation on the interactions with beneficial insects to better assess their utility for pest control and productivity in agriculture.

## 4. Materials and Methods

### 4.1. Study System

As an agriculturally relevant model plant, we used cultivated tomato, *Solanum lycopersicum* L. (variety Moneymaker), a perennial species grown annually for food production. The insect species employed included the Egyptian cotton leafworm, *Spodoptera littoralis* (Lepidoptera: Noctuidae), a generalist herbivore and a pest in many food crops [41]; and the spined soldier bug, *Podisus maculiventris* (Hemiptera: Pentatomidae), a generalist predator with a broad host range [42]. The bacterial strains used in this study are the two rhizosphere isolates *Pseudomonas simiae* WCS417r (formerly *Pseudomonas fluorescens* WCS417r) and *Pseudomonas fluorescens* 89B61, two strains with well-described ability to trigger systemic resistance in tomato plants [16,22,23]. The strains were kindly provided by Corné Pieterse, University of Utrecht (WCS417r) and Joe Kloepper, Auburn University (89B61).

### 4.2. Cultivation of Study Organisms

Plants were grown in 1 L pots filled with autoclaved (2 cycles of 21 min at 121 °C) potting soil (Substrat 2, Klasmann-Deilmann GmbH, Geeste, Germany). Tomato seeds were germinated on water agar and planted in a tray filled with sterilized potting ground soil. Seedlings were transplanted to pots and grown in a climate chamber (24 °C/22 °C day/night, 60–70% r.h. and L16:D8) until used for bioassays (4–5 weeks old plants in their vegetative stage).

Eggs of *S. littoralis* were obtained from Syngenta AG (Stein, Switzerland) and larvae reared on artificial diet (General Purpose Lepidopteran Diet, Frontiers Scientific, USA) in a climate chamber (24–27 °C, 60–70% r.h., L16:D8). We used 3-day old caterpillars for our experiments. *P. maculiventris* was reared in a climate room (27 °C, 60–70% r.h., L16:D8) in cages containing a *Brassica oleracea* L. var. *gemmifera* cv. Cyrus (Brussels sprouts). Young nymphs were fed on *Ephestia* eggs (provided by Andermatt Biocontrol, Andermatt, Switzerland) or early instars of *Pieris brassicae* larvae (from a laboratory colony), while later instars and adults primarily fed on mealworms *Tenebrio molitor* (Profiterr Schreiber, Widen, Switzerland). Insects were used for the experiments when they had reached the adult stage.

Rhizobacteria were grown for 2 days on King’s B medium (KB) agar plates containing chloramphenicol (13 μg mL^−1^), ampicillin (40 μg mL^−1^), and cycloheximide (100 μg mL^−1^) at 24 °C. Each strain was then grown overnight in 10 mL of Lysogeny broth (LB) at 24 °C on a rotatory shaker (180 r.p.m.), then 200 µL of the broth was evenly spread on KB agar plates (without antibiotics) and grown for an additional 24 h at 24 °C. Prior to inoculation in sterile soil, bacterial cells were collected in 10 mL 10 mM MgSO_4_, centrifuged (10 min, 1697 r.c.f), washed once with 10 mM MgSO_4_, re-suspended in sterile H_2_O, and adjusted to an OD_600_ = 0.1. Tomato plants were inoculated one day after transplantation to pots by adding 32.5 mL of a given bacterial suspension to the soil. To establish that root inoculation consistently resulted in colonization levels necessary to trigger resistance (>10^5^ CFU/g of root) [43], we assessed root colonization of at least three randomly selected rhizobacteria-inoculated plants in different experiments by washing roots and performing serial dilution plating on KB agar (with antibiotics). To establish that mock-inoculated control plants were generally free of *Pseudomonas simiae* and *Pseudomonas fluorescens*, we followed the same procedure and assessed root colonization of at least three randomly selected mock-inoculated control plants in different experiments. *Pseudomonas* bacteria were only occasionally found on roots of control plants.

### 4.3. Plant Treatments

To investigate the effects of each rhizobacteria strain on plant biomass and on direct and indirect resistance, we subjected plants to three rhizobacteria treatments (inoculation with *Pseudomonas simiae*, inoculation with *Pseudomonas fluorescens*, or mock inoculation), each of which was tested with and without herbivory (six treatments in total). Herbivore treatments in plant volatile collections and behavioral assays entailed placing a clip cage on the first and second true leaf, each enclosing two *S. littoralis* larvae. For analyses of phytohormones and alkaloids, one larva was similarly caged on a single leaf. In each case, treatments without herbivores received empty clip cages as a control.

### 4.4. Rhizobacteria Effects on Plant Biomass and Direct Resistance

Caterpillar performance: To investigate the effects of inoculation with *P. simiae* and *P. fluorescens* on plant resistance, the performance of *S. littoralis* was assessed by recording larval weights after a 7-day feeding period. For this, each plant (10 replicates per treatment) received four larvae while the plant was enclosed by a glass dome to prevent larvae from escaping. Constant airflow was applied to avoid condensation within the dome and larvae were allowed to move freely on the plants.

Plant biomass: Fresh shoot biomasses of plants of all treatments were recorded during one experiment (see Section 4.5) to assess potential effects on plant biomass promotion.

Phytohormones and alkaloid compounds: To evaluate effects of rhizobacteria inoculation on the main mechanisms underlying resistance to pests in tomato plants, we quantified alkaloids and levels of the phytohormones jasmonic acid (JA) and salicylic acid (SA) on plants subjected to the different treatments. Leaf tissue of 5–12 plants per treatment was frozen in liquid nitrogen, lyophilized, and ground to powder in a 2010 Geno/grinder^®^ (SPEX^®^ SamplePrep, Metuchen, NJ, USA). From the homogenized tissue, 10–12 mg was weighed into an Eppendorf^®^ tube for subsequent extractions.

Phytohormones and alkaloid compounds were extracted from the same leaf material following a modified protocol [44] by adding 650 µL of methanol, 750 µL of ammonium acetate (10 mM), and 50 µL of isotope-labelled standards solution containing d5-JA and d4-SA. Sample tubes were vortexed for 20 s, sonicated for 15 min, and then centrifuged for 10 min (20,000 rcf, RT). One milliliter of supernatant was transferred to a new 2 mL Eppendorf^®^ tube and dried down in a Savant Speed Vac Concentrator SPP1010. Samples were resuspended in 100 µL of 0.1% formic acid solution and vortexed for 30 s. Sample tubes were subsequently incubated on ice for 10 min and then centrifuged at 20,000 rcf for 10 min. Resulting extracts were analyzed by liquid chromatography–mass spectrometry (Agilent 6550 iFunnel Q-TOF LC/MS) with a RRHD Zorbax Eclipse Plus-C8 column (100 mm length, 2.1 mm diameter, 1.8 μm particle size). For phytohormone analysis, we used a solvent gradient of ultrapure water supplemented with 5 mM ammonium formate (A)—acetonitrile (B). Initial conditions were 99% A for 0.7 min, increasing linearly to 95% B over 5.6 min and held for another 3.7 min at 95% B with a flow rate of 0.4 mL/min. JA and SA were identified and quantified based on known concentrations of the labeled standards.

For alkaloid analysis, we used a solvent gradient of ultrapure water supplemented with 0.1% formic acid (A)—acetonitrile (B). Initial conditions were 99% A for 0.7 min, increasing linearly to 98% B over 7.3 min, then an additional 2 min at 98% B with a flow rate of 0.4 mL/min. The auto-sampler was cooled to 4 °C and the column temperature was set to 50 °C. In the MS, the liquid effluent was ionized by electrospray ionization in positive (alkaloids) or negative (phytohormones) mode. Four different tomatidine-related alkaloids and five different solasodine-related alkaloids were tentatively identified by scanning for [M+H]^+^ *m*/*z* 416.35 (tomatidine) and *m*/*z* 414.33 (solasodine) and quantified based on peak area.

### 4.5. Rhizobacteria Effects on Plant Indirect Resistance

**Figure 4:**
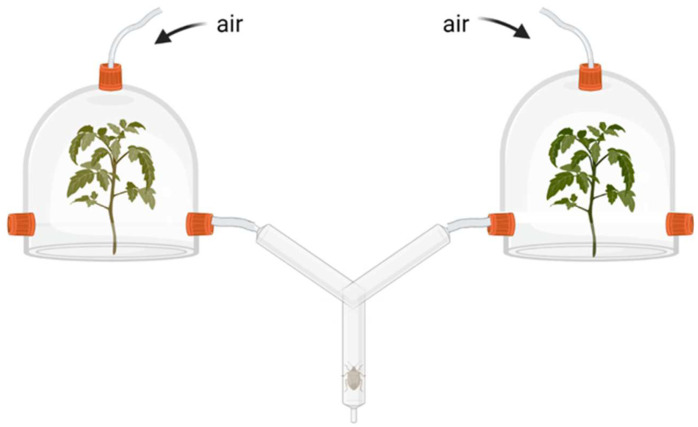
Schematic representation of the Y-tube olfactometer used in the behavioral assays with the predators.

Behavioral responses of predatory bugs: To test whether inoculation with one of the rhizobacteria strains affects the behavioral responses of the predatory bug *P. maculiventris* to tomato plants, we conducted bioassays with a dynamic air-flow Y-tube olfactometer (each arm 15 cm, ø 2.5 cm) (Figure 4) [10,45]. Adult *P. maculiventris.* were given pairwise choices between odor sources (mock- vs. rhizobacteria-treated), including clean air along with volatiles from plants exposed to one of the six treatments described above. We used Teflon tubing to connect each of the two side arms of the olfactometer to a glass dome harboring the odor source. Before reaching the glass container, the pressurized air was filtered through activated charcoal, and the airflow was adjusted to 2.5 L min^−1^. In tests where plants were used, a single plant was introduced in each glass dome. A Teflon plate separated the plant pot containing the soil from the aboveground biomass; thus, bugs have been exposed only to the odors of the plant foliage. The Y-tube olfactometer was placed vertically and the glass domes containing the odor sources were kept behind a panel covered with aluminum foil that prevented insects from visually detecting the plants. Predators were introduced individually at the downwind end of the entry arm and observed until they walked up at least two-thirds of one of the side arms (10 out of 15 cm); bugs that did not make a choice within 5 min were recorded as ‘no response’ and excluded from statistical analyses. The position (right/left) of odor sources was switched after every trial. Experiments were carried out between 10:00 and 16:00 h in a climate room (25 ± 2 °C, 50–70% r.h.) with light bulbs (80 µmol m^−2^ s^−1^) above the olfactometer and the containers with the odor sources. All bugs used in Y-tube olfactometers were starved 16 h prior to the start of the experiment. Five to 10 bugs were tested per pair of plant with 6 to 8 replicates of each plant pair. To ensure that bugs perceive and respond to plant odors in our system, a comparison between *S. littoralis*-infested control plant odors and clean air was included in the experiment.

*Plant volatile emissions:* To evaluate the effects of plant inoculation with *P. simiae* and *P. fluorescens* on constitutive and herbivore-induced plant volatile emissions, we collected plant volatiles from tomato plants in a dynamic headspace collection system [46]. Each plant was placed in a climate-controlled collection room (L16:D8, 25–27 °C) under sodium-halogen lamps (100–120 µmol m^−2^ s^−1^) two days prior to the volatile collection, when 50% of the plants were also infested with caterpillars of *S. littoralis*. On the collection day, all larvae were removed from herbivore treatments and plants were placed under glass domes (~10 L). Aboveground plant parts were separated from the soil by Teflon plates fitted around the plant stem to limit the influence of non-plant odor sources on the analyzed plant bouquet. Plant volatiles were collected (for 7 h—from 09:00 to 16:00) onto HayeSep Q adsorbent filters by pumping charcoal-purified air into the top of the glass dome at a rate of 1.2 L min^−1^ and simultaneously applying a vacuum of 0.9 L min^−1^ to the back of the filter that was positioned in one of the glass dome side outlets. We had three experimental blocks, each performed one week apart, and each including 5 biological replicates of each of the 6 treatments, adding to 15 replicates per treatment. We also collected volatiles from empty glass domes on each of the experimental days to determine background volatile compounds and had in total 6 replicates.

Volatiles were analyzed by gas chromatography (GC) coupled to a mass spectrometer (MS) and to a flame ionization detector (FID) (Agilent 7890B GC, Agilent 5977A MSD, Basel, Switzerland). After each collection, plant volatiles were eluted from HayeSep Q adsorbent filters with 150 µL dichloromethane. Measures of 2 µL were transferred in splitless mode to the analytical column (30 m × 0.25 mm ID, 0.25 μm film thickness, HP-5MS UI, Agilent Technologies, Basel, Switzerland). The temperature of the oven was initially held at 35 °C for 30s and subsequently ramped to 280 °C in 8 °C/minute intervals. An electron impact ionization at 70 eV was used to ionize the column effluent. Mass scanning was carried out from *m/z* 40 to 350 with 4.5 scans s^−1^.

GC–MS data were processed using the MetAlign–MSClust bioinformatic pipelines [47]. MetAlign corrects the baseline, eliminates noise and resolves co-elution issues of each GC–MS output file, subsequently aligning the individual mass peaks of all chromatograms. MSClust then clusters the aligned mass peaks and reconstructs mass spectra of the individual volatile organic compounds (VOCs) [48]. Only mass peaks with a retention time within 5–36 min and in the 55–400 *m/z*-range were further processed. VOCs were annotated by comparing their mass spectra with those of the reference databases NIST (National Institute of Standards and Technology, Gaithersburg, MD, USA), Wiley libraries, and the Wageningen Mass Spectral Database of Natural Products, and by comparing the calculated AI with those in the literature. For this, a standard mixture of linear alkanes (C7–C25) was also analyzed by GC-MS for the determination of the arithmetic retention index (AI) values of the VOCs. Analysis of the alkane mixture was performed under the same conditions as described above. Peak height of individual compounds was normalized by fresh shoot biomass (g) and compounds were included in the analyses if they were detected in a minimum of 50% of the samples of a given treatment.

### 4.6. Data Analysis

Following normality tests, plant biomass data were analyzed with a generalized linear (mixed) model (GLMM) with a gamma distribution and a log link function. Herbivory and inoculation were included in the model as fixed factors as well as their interaction. Caterpillar biomass data were analyzed with a generalized linear model (GLM) with a gamma distribution and a log link function and treatment, plant replicate, and their interaction were included in the model as fixed factors. Phytohormone data were BoxCox-transformed and analyzed with a one-way ANOVA using inoculation as a fixed factor or a two-way ANOVA (type II) with herbivory, inoculation, and their interaction as fixed factors. One sample was removed as an outlier from the phytohormone analyses due to an extreme value. Alkaloid data were analyzed using a generalized least-squares model with a constant variance function to account for heterogeneity of variances and herbivory, inoculation, and their interaction as fixed factors. Behavioral responses in the olfactometer assay were analyzed using a binomial test with average per plant-pair. The composition of volatile blends was analyzed with cluster analysis and a heatmap was used for data visualization (MetaboAnalyst 4.0). Data were normalized by the median, log transformed, and range scaled. Clustering was done using Euclidian distances, and Ward’s criterion as clustering method.

## Data Availability

All data shown in this study are available in the article or Supplementary Materials.

## Supplementary Materials

The following supporting information can be downloaded at: https://www.mdpi.com/article/10.3390/plants11070920/s1, Figure S1: Fresh shoot biomass (g) of tomato plants *Solanum lycopersicum* exposed inoculation with two strains of rhizobacteria and herbivory by *Spodoptera littoralis*, Table S1: Volatile organic compounds quantified in blends of tomato plants *Solanum lycopersicum* exposed to two strains of rhizobacteria and herbivory by *Spodoptera littoralis*.
