# Supplementary material for: Negative Effects of Rhizobacteria Association on Plant Recruitment of Generalist Predators
Source: Plants (Basel). 2022 Mar 29;11(7):920. doi: 10.3390/plants11070920 (PMC9003080; doi:10.3390/plants11070920)
Supplement: Supplementary file 1 [file plants-11-00920-s001.zip › plants-1487842-supplementary.pdf]

Supplementary Materials

Negative effects of rhizobacteria association on plant recruitment of generalist predators

Tobias B. Löser [1,†], Dani Lucas-Barbosa [1,2,†], Monika Maurhofer [1], Mark. C. Mescher [1] and Consuelo M. De Moraes [1,*]

[1] Department of Environmental System Sciences, ETH Zürich, 8092 Zürich, Switzerland; tobias.loeser@usys.ethz.ch, monika.maurhofer@usys.ethz.ch, mescher@usys.ethz.ch, consuelo.demoraes@usys.ethz.ch

[2] current address: Vector Entomology Group, Institute of Parasitology, Vetsuisse Faculty, University of Zürich, Switzerland; dani.lucas-barbosa@vetparas.uzh.ch

[*] Correspondence: consuelo.demoraes@usys.ethz.ch

[†] These authors contributed equally to this work.

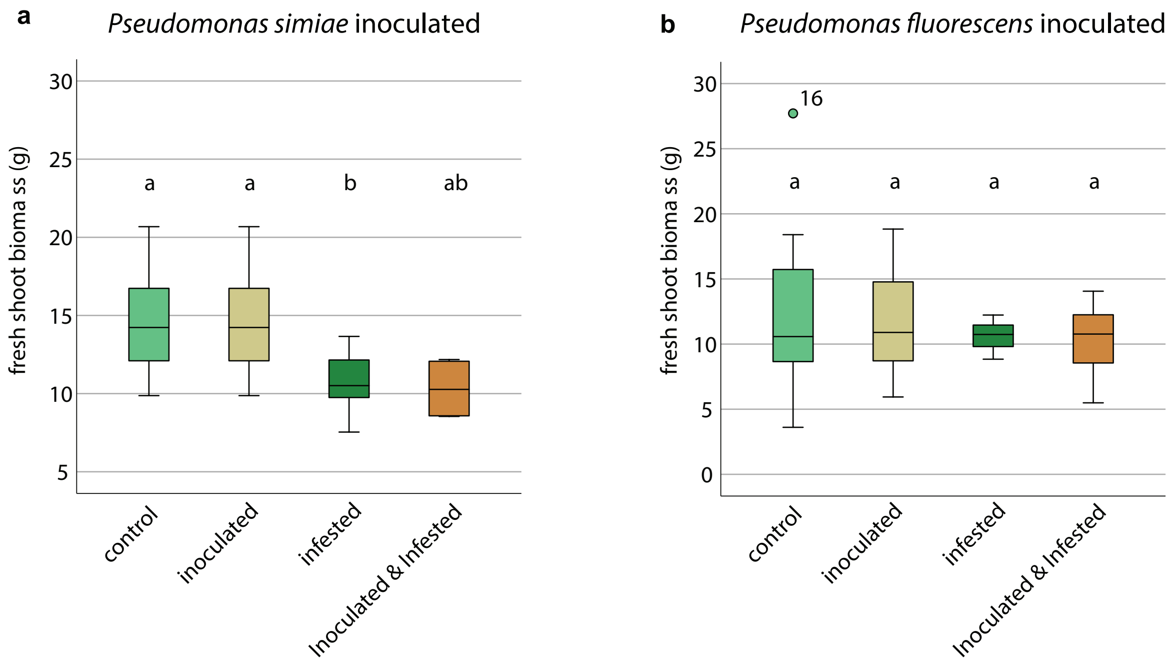

Figure S1. Fresh shoot biomass (g) of tomato plants *Solanum lycopersicum* exposed to inoculation with two strains of rhizobacteria and herbivory by *Spodoptera littoralis*: (a) non-infested control plants; plants inoculated with *Pseudomonas simiae*; herbivore-infested plants; infested and inoculated plants with *Pseudomonas simiae*. (b) non-infested control plants; plants inoculated with *Pseudomonas fluorescens*; infested plants; plants infested and inoculated with *Pseudomonas fluorescens*. Data was analyzed with generalized linear mixed model, and Bonferroni used as *post-hoc* test.

Table S1: Volatile organic compounds quantified in blends of tomato plants *Solanum lycopersicum* exposed to two strains of rhizobacteria and herbivory by *Spodoptera littoralis* adding to 6 treatments: (1) non-infested control plants (C); (2) inoculated plants with *Pseudomonas fluorescens* (Pf) or (3) with *Pseudomonas simiae* (Ps); (4) herbivore-infested plants (H); (5) infested and inoculated plants with *Pseudomonas fluorescens* (PfH) or (6) infested and inoculated with *Pseudomonas simiae* (PsH).

Volatile compounds	AI*	non-infested<br>Peak height / g FW<br>mean ± SD	infested<br>Peak height / g FW<br>mean ± SD	*P. flurorescens* inoculated<br>Peak height / g FW<br>mean ± SD	*P. flurorescens* inoculated & infested<br>Peak height / g FW<br>mean ± SD	*P. simiae* inoculated<br>Peak height / g FW<br>mean ± SD	*P. simiae* inoculated & infested<br>Peak height / g FW<br>mean ± SD
Monoterpenoids							
α-Pinene	939	495 ± 424	499 ± 441	616 ± 338	479 ± 237	539 ± 295	503 ± 240
Cyclohepta-1,3,5-triene, 3,7,7-trimethyl-	976	688 ± 645	700 ± 681	690 ± 463	654 ± 377	735 ± 464	490 ± 305
β-Pinene	984	24 ± 26	26 ± 27	25 ± 26	26 ± 22	31 ± 19	45 ± 67
Myrcene	992	673 ± 401	916 ± 616	708 ± 405	734 ± 391	677 ± 408	715 ± 329
2-Carene	1007	19686 ± 12198	18919 ± 11249	21901 ± 9613	19535 ± 7934	22780 ± 13065	20762 ± 8066
α-Phellandrene	1010	1017 ± 875	1130 ± 1352	1150 ± 832	956 ± 674	885 ± 526	944 ± 466
α-Terpinene	1022	435 ± 357	486 ± 591	461 ± 321	388 ± 295	333 ± 188	433 ± 254
p-Cymene	1031	587 ± 472	675 ± 583	750 ± 481	587 ± 313	602 ± 336	598 ± 322
β-Phellandrene	1037	7145 ± 5045	7340 ± 5529	8204 ± 4407	7067 ± 3642	7394 ± 4127	6837 ± 2673
β-Ocimene, (*E*)-	1064	99 ± 115	322 ± 343	142 ± 146	248 ± 246	125 ± 129	190 ± 125
Terpinolene	1095	13 ± 18	18 ± 47	18 ± 25	7 ± 17	12 ± 15	19 ± 44
Linalool	1102	8 ± 11	96 ± 140	2 ± 5	72 ± 161	3 ± 5	46 ± 64
Dihydrocarvone, (Z)-	1186	17 ± 30	15 ± 27	29 ± 35	31 ± 31	14 ± 19	11 ± 17
Anetofuran	1196	67 ± 52	74 ± 56	90 ± 73	78 ± 65	62 ± 48	97 ± 89
Sesquiterpenoids							
γ-Elemene	1474	697 ± 600	759 ± 622	888 ± 629	643 ± 441	512 ± 620	752 ± 667
α-Copaene	1391	784 ± 631	731 ± 532	789 ± 463	972 ± 702	806 ± 648	763 ± 569
β-Caryophyllene	1440	1622 ± 1187	1435 ± 913	1760 ± 1016	1385 ± 797	1179 ± 838	1365 ± 1033
Guaia-6,9-diene	1459	30 ± 26	22 ± 25	39 ± 30	20 ± 18	18 ± 17	32 ± 26
α-Humulene	1474	510 ± 368	471 ± 272	551 ± 303	451 ± 235	377 ± 257	439 ± 311
β-Ionone, (*E*)-	1499	47 ± 64	161 ± 205	169 ± 350	233 ± 432	77 ± 141	204 ± 300
Caryophyllene oxide	1609	32 ± 29	22 ± 18	38 ± 29	31 ± 19	27 ± 18	23 ± 18
Homoterpene							

Trideca-1,3,7,11-tetraene, 4,8,12-trimethyl, (*E,E*)-	1585	10104 ± 9762	12766 ± 12958	9816 ± 10900	13616 ± 15845	7543 ± 6761	9290 ± 9240
Fatty acid and amino acids derivatives							
2-Ethyl-1-hexanol	1029	476 ± 339	589 ± 644	455 ± 318	446 ± 394	376 ± 267	509 ± 449
3-Hexen-1-ol, butanoate, (*Z*)-	1189	0 ± 0	165 ± 204	57 ± 218	182 ± 443	0 ± 0	77 ± 177
N containing compound							
3-Methylbutanal, O-methyloxime-**		16 ± 13	13 ± 9	31 ± 50	17 ± 13	14 ± 11	24 ± 14
Unknown compound							
unknown m/z 168	1337	17 ± 18	24 ± 34	22 ± 18	16 ± 17	17 ± 15	19 ± 17

* Calculation of Arithmetic Index (AI) as described by Adams (2001)

** We did not calculate the arithmetic index for this compound because the shortest chain linear hydrocarbon we detected was octane
